# Over-Expression of *SlSHN1* Gene Improves Drought Tolerance by Increasing Cuticular Wax Accumulation in Tomato

**DOI:** 10.3390/ijms151119499

**Published:** 2014-10-27

**Authors:** Ayed M. Al-Abdallat, Hmoud S. Al-Debei, Jamal Y. Ayad, Shireen Hasan

**Affiliations:** 1 Department of Horticulture and Crop Science, Faculty of Agriculture, the University of Jordan, Amman 11942, Jordan; E-Mails: debeih@ju.edu.jo (H.S.A.-D.); ayadj@ju.edu.jo (J.Y.A.); 2 Hamdi Mango Center for Scientific Research, the University of Jordan, Amman 11942, Jordan; E-Mail: s.hasan@ju.edu.jo

**Keywords:** bioinformatics, cuticle, drought, *Solanum lycopersicum*, transcription factor

## Abstract

Increasing cuticular wax accumulation in plants has been associated with improving drought tolerance in plants. In this study, a cDNA clone encoding the SlSHN1 transcription factor, the closest ortholog to *WIN/SHN1* gene in *Arabidopsis*, was isolated from tomato plant. Expression analysis of *SlSHN1* indicated that it is induced in response to drought conditions. The over-expression of *SlSHN1* in tomato under the control of the constitutive *CaMV 35S* promoter produced plants that showed mild growth retardation phenotype with shiny and dark green leaves. Scanning electron microscopy showed that the over-expression of *SlSHN1* in tomato resulted in higher cuticular wax deposition on leaf epidermial tissue when compared to non-transformed plants. Expression analysis in transgenic lines over-expressing *SlSHN1* indicated that several wax-related synthesis genes were induced. Transgenic tomato plants over-expressing *SlSHN1* showed higher drought tolerance when compared with wild type plants; this was reflected in delayed wilting of transgenic lines, improved water status and reduced water loss rate when compared with wild type plants. In conclusion, the *SlSHN1* gene can modulate wax accumulation and could be utilized to enhance drought tolerance in tomato plant.

## 1. Introduction

In many parts around the world, drought is considered a major constraint that affects plant growth and development. To overcome this limitation and to improve crop productivity, it is important to improve the drought tolerance associated traits in cultivated plants [1]. In nature, plants have developed different physiological and biochemical mechanisms to adapt to drought conditions [2]. Such mechanisms include stomata closure, epidermal wax deposition, accumulation of osmolytes, such as late embryogenesis abundant proteins, and growth retardation [3]. From this perspective, the identification of key regulatory genes may offer the opportunity to breed new varieties with improved adaption to drought conditions [4]. To achieve this, several studies were conducted to understand the mechanisms by which plants perceive environmental signals and transmit the signals to cellular machinery that activates adaptive responses (reviewed in [5,6]).

An important step in controlling plant abiotic stress tolerance responses appears to be the transcriptional activation or repression of stress-responsive genes [4,7]. Transcriptional regulation of gene expression is largely mediated by the specific recognition of *cis*-acting promoter elements by *trans*-acting sequence specific DNA-binding proteins known as transcription factors (TFs). These TFs belong to several families, such as AP2/ERF, bZIP, NAC, MYB, MYC, zinc-finger and WRKY [4,7,8]. In many instances, the over-expression of these factors or their active forms in plants confers drought tolerance phenotypes [9,10].

In plants, the cuticle layer plays a major role in improving internal water status by reducing nonstomatal water transpiration [11,12]. The waxy cuticle layer of leaves serves to inhibit water loss and thus decrease dehydration of underlying cells [13]. Several studies were conducted to understand the mechanisms by which plants synthesize waxy components of the cuticle in the epidermis [14,15], and several regulatory proteins including TFs were found to activate large numbers of cutin and waxes biosynthesis genes [16,17,18]. In many instances, the over-expression of these regulatory genes resulted in the activation of numerous wax synthesis genes and the production of a thick cuticle layer [17]. For instance, homeodomain-leucine zipper TF HDG1 was found to play a major role in the induction of several cutin biosynthesis genes in rice and *Arabidopsis* plant [19]. Similarly, the *HDG1* homologous tomato gene, *CUTIN DEFICIENT2* (*CD2*), was found to play a major role in regulating cutin synthesis in fruit and other organs [20]. Several MYB TF were found to enhance or repress wax formation in response to abiotic stresses [17]. For instance, two MYB TFs from *Arabidopsis*, AtMYB41 and AtMYB96, were found to enhance wax deposition and were considered positive regulators of cuticle biosynthesis [21]. The stress-responsive TF MYB96 was found to play a positive role in the induction of the expression of several wax synthesis genes in *Arabidopsis* [22]. Contrary to AtMYB96, the stress-responsive TF MYB41 was found to act as a repressor of several cutin biosynthesis genes [23].

One of the first transcription factors identified as a cuticle biosynthesis regulator was the AP2 domain-containing WAX INDUCER1/SHINE1 (WIN1/SHN1) [24,25]. The *SHN1/WIN1* gene was characterized in *Arabidopsis* plant where it was found to improve water use efficiency by modifying leaf diffusive properties due to accumulation of high levels of wax. The *Arabidopsis SHN1/WIN1* gene was found to coordinate the gene expression of a large number of enzymes that are involved in the elongation of fatty acids and the formation of aliphatic compounds [26,27]. Gene expression profiling of *SHN1/WIN1* loss of function mutants was found to affect many genes involved in wax biosynthesis [26]. Further analyses indicate that the *Arabidopsis SHN1/WIN1* loss of function mutant had increased transpiration rates relative to wild type plants [24,25]. In addition, the over-expression of *SHN1/WIN1* in *Arabidopsis* plant resulted in improved drought tolerance and reduced transpiration rate [28,29]. The over-expression of *AtSHN2*, a close homolog to *SHN1/WIN1*, in rice plants resulted in improved cellulose biosynthesis with reduced lignin production [30]. In addition, the functional characterization of the *SHN1/WIN1* orthologous gene in barley indicates a major role in cutin biosynthesis and the formation of adhering hulled caryopsis type [31]. Recently, the functional characterization of tomato *SlSHN3* showed an important role in regulating wax biosynthesis and fruit cuticle formation [28]. The over-expression of *SlSHN3* in *Arabidopsis* plants produced similar phenotypes to *SHN1/WIN1* over-expressing plants and conferred dehydration tolerance in harvested tomato fruit. Meanwhile, loss of function mutation and gene silencing of *SlSHN3* in tomato plants resulted in reduced levels of cuticular wax and altered fruit morphology. Furthermore, gene silencing and loss of function mutation *SlSHN3* resulted in higher postharvest water loss rates and enhanced susceptibility to fungal infection [28,32].

In this study, the characterization of the closest *SHN1/WIN1* orthologous gene in tomato, *SlSHN1*, and its role in mediating drought tolerance is described. The deduced amino acid sequence of the SlSHN1 protein showed high level of similarity to the *Arabidopsis* SHN1/WIN1 protein. The *SlSHN1* gene was induced in response to drought conditions indicating a potential role in mediating tolerance to dehydration stress. Transgenic tomato plants over-expressing *SlSHN1* showed reduced water loss rate and enhanced growth under drought conditions. In addition, the expression of wax biosynthesis genes was found to be induced in the over-expressing *SlSHN1* lines.

## 2. Results and Discussion

### 2.1. Isolation of WIN1/SHN1 Orthologous Gene in Tomato

To identify *WIN1/SHN1* orthologous gene in tomato, a bioinformatics analysis was carried out based on the full-length sequence data of the previously described *WIN1*/*SHN1* gene in *Arabidopsis* (GenBank accession number: AAR20494.1). For this purpose, a TBLASTN search was performed against the annotated ITAG2.3 predicted tomato cDNA sequences database [33]. As a result, a full length cDNA sequence was retrieved and found to encode a putative ethylene-responsive transcription factor (*Solyc03g116610*: GenBank ID: XM_004235917). The deduced complete sequence (202 amino acids) of Solyc03g116610 has 65% identity with the WIN1/SHN1 protein and 91% with *Sotub03g030060.1.1* from potato (PGSC0003DMT400001703; PGSC DM 3.4 Release [34]).

Using a gene-specific primer pair designed from the XM_004235917 nucleotide sequence, a 609 bp fragment containing the coding sequence of *Solyc03g116610* was cloned from leaf tissues of two weeks old tomato seedlings. The *Solyc03g116610* coding sequence was previously identified by *in silico* analysis in Shi *et al.* [28] and was described as *SlSHN1* with two other additional tomato members *SHN-like* genes, *SlSHN2* and *SlSHN3*. The Shi *et al.* [28] study concentrated on *SlSHN3*, which is the closest ortholog to *AtSHN3* (*At5g25390*) in *Arabidopsis*, and its role in tomato fruit cuticle formation and dehydration stress tolerance in fruits. In this study, the functional characterization of *SlSHN1*, the closest ortholog to the previously characterized *WIN1*/*SHN1* in *Arabidopsis*, and its role in mediating drought tolerance in tomato with particular emphasis on vegetative tissue is investigated.

Mining the sequence data of the released tomato genome (Tomato Genome Consortium 2012) in the Sol Genomics Network [34] and the MIPS tomato genome database [35] indicated that the *SlSHN1* gene is located on the lower arm of chromosome 3 (approximate position 60.01 MB) and it is annotated as *Solyc03g116610*. One splice variant (*Solyc03g116610.1*) was predicated to encode the functional SlSHN1 protein (203 amino acids). In addition, the SNP marker “*solcap_snp_sl_34148*” was identified in the coding sequence of *Solyc03g116610* (data not shown). The genomics structure of the *Solyc03g116610*.1 variant indicates that *SlSHN1* cDNA sequence length is 1100 bps and contains two exons and one intron (118 bp), a 147 bp 5'-UTR and a 225 bp 3'-UTR. The *SlSHN1* gene structure was similar to *WIN1*/*SHN1* [24,25], *HvNud* [31] *OsWR1* [36] and *SlSHN3* [28] where all of them contained a single intron and two exons structure indicating a conserved function.

**Figure 1:**
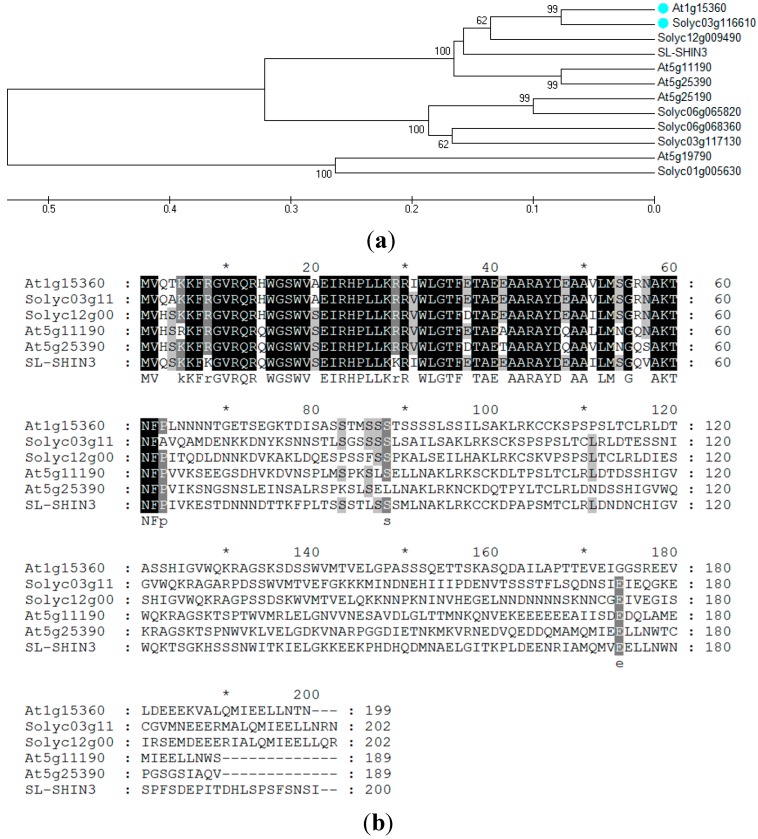
(**a**) Phylogenetic analysis of *Arabidopsis* proteins belonging to the ERF Gene Family Group Va and (**b**) their closest ortholog in tomato. Multiple sequence alignment analysis of three selected WIN/SHN *Arabidopsis* proteins (At1g15360 (WIN1/SHN1), At5g11190 (WIN2/SHN2) and At5g25390 (WIN3/SHN3)) with their closest ortholog in tomato (SlSHN1 (Solyc03g116610), SlSHN2 (Solyc12g009490) and SlSHN3) [28]. The AP2 domain is underlined. White letters shaded indicate amino acids that are either 100% identical (black) or identical in at least 80% (dark gray) or identical in at least 60% (light gray) of all proteins.

The domain features of the SlSHN1 protein indicated the presence of an AP2 domain (Pfam I.D.: PF00847; [37] involved in DNA binding) in the *N*-terminal region in the protein belonging to the ethylene responsive factors (ERF) superfamily [38] (Figure 1a). Following the previously described grouping of ERF-based domain architecture of ERF proteins in *Arabidopsis* and rice [38], the SlSHN1 protein was found to belong to Group V and in particular to subgroup Va. This subgroup is characterized for having two motifs known as CMV-1 and CMV-2 in the *C*-terminal region of its members, which distinguish them from members of subgroup Vb.

Phylogenetic analysis of *Arabidopsis* proteins belonging to ERF Gene Family Group V (subgroup a) [38] and their ortholog in tomato (retrieved by BLAST search against ITAG2.3 predicted tomato protein sequences database [34]) clustered the SlSHN1 protein with WIN1/SHN1 (Figure 1b). This sub-group contains members known to regulate the accumulation of epidermal wax in plants [24,25,28,31]. The MSA and phylogenetic analysis were in general agreement with the results of Shi *et al.* [28] placing SlSHN1 as the closest ortholog to WIN1/SHN1.

### 2.2. SlSHN1 Expression in Response to Drought Conditions

**Figure 2:**
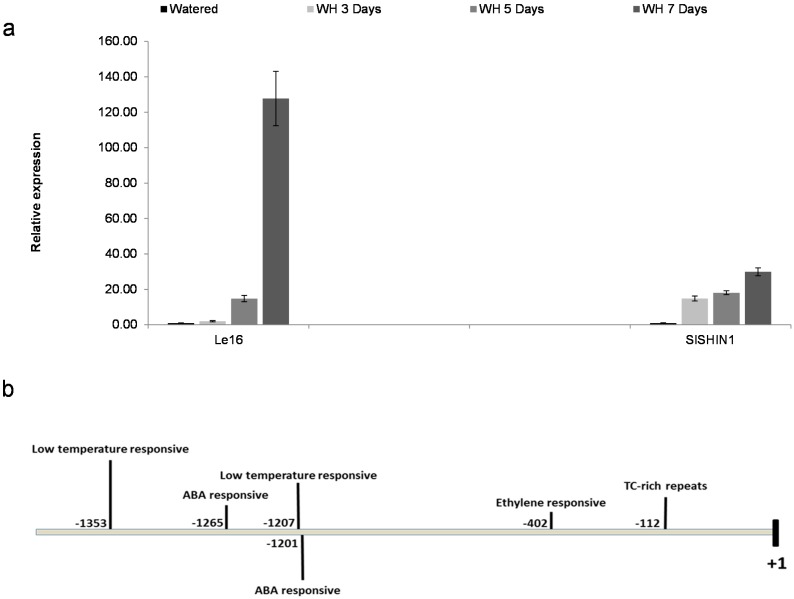
(**a**) Gene expression analysis of *SlSHN1* in response to drought conditions. Tomato Moneymaker plants were subjected to water withholding (WH) conditions for 3, 5 and 7 days and compared to well-watered plants (control). The stress responsive *Le16* (*Solyc10g075090*) gene was included as a control. The bars are standard deviations (SD) of three technical repeats; (**b**) Characteristics of the promoter region of *SlSHN1* in tomato. Distribution of major known stress-related *cis*-elements in the first 1500 bp of the *SlSHN1* promoter region as identified using PlantCARE *cis*-element databases.

The expression patterns of *SlSHN1* in response to drought treatment (water withheld for 3, 5 and 7 days) in Moneymaker plants were analyzed using quantitative real-time PCR (RT-qPCR). As a positive control, the gene expression patterns of the stress-inducible gene, *Le16* (*Lycopersicon esculentum protein 16*; *Solyc10g075090*), encoding a phospholipid transfer protein from tomato were analyzed [39]. The *Le16* gene is known to be highly induced in response to drought stress in tomato plant. As expected, the expression of the *Le16* marker gene was induced in response to drought treatment after 5 days and was at the highest level after 7 days of stress (Figure 2a). The expression of *SlSHN1* was induced slightly after three days of drought and showed higher levels of induction after seven days of stress when compared with wild type plants under normal conditions (Figure 2a). This is in general agreement with induction patterns of *OsWR1*, the closest rice ortholog to *SlSHN1*, where its transcripts levels increased gradually after drought treatment [36]. In addition, the observed expression pattern of *SlSHN1* in response to drought was similar to the patterns observed for *WXP1* in drought treated *Medicago truncatula* [40].

In order to identify *cis*-acting elements involved in abiotic responses in the promoter region of *SlSHN1*, the 1500 bp upstream sequence from the 5'-UTR region start (retrieved from [34]) was mined using the PlantCARE *cis*-element databases [41]. Using this strategy, several stress responsive elements were identified in the *SlSHN1* promoter region, including: the *ABRE* (*Abscisic Acid Responsive Element*), *LTR* (*low temperature responsive*) and *TC-rich repeats* (Figure 2b).

### 2.3. Over-Expression of SlSHN1 in Tomato Affects Its Growth and Improves Drought Tolerance

**Figure 3:**
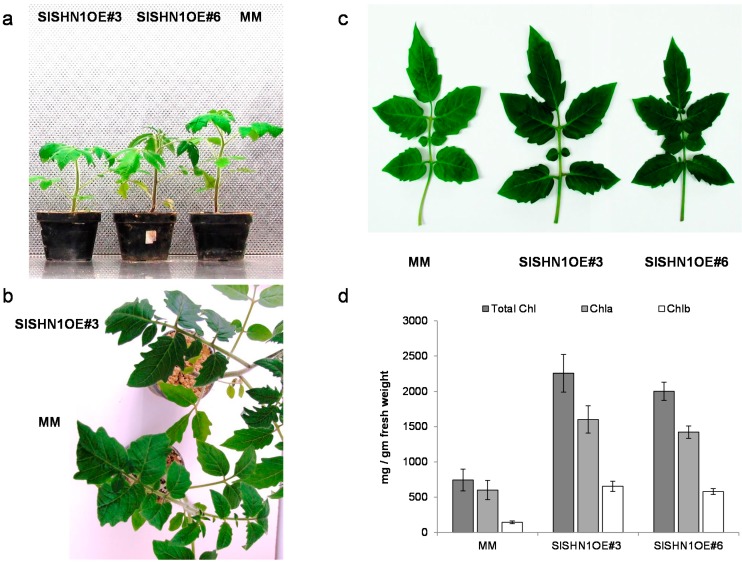
(**a**) Growth performance of two-week old tomato seedlings of two transgenic lines over-expressing *SlSHN1* (*SlSHN1OE#3* and *SlSHN1OE#6*) and wild type (MM) under normal conditions; (**b**) The shiny appearance of the transgenic line over-expressing *SlSHN1* (*SlSHN1OE#3*) compared with wild type plant; (**c**) The dark green color phenotype of leaves in transgenic lines over-expressing *SlSHN1* (*SlSHN1OE#3* and *SlSHN1OE#6*) compared with wild type leaf (MM); (**d**) Total chlorophyll (total Chl), Chlorophyll a (Chla) and Chlorophyll b (Chlb) content in two transgenic lines over-expressing *SlSHN1* (*SlSHN1OE#3* and *SlSHN1OE#6*) and wild type leaves (MM).

To test whether *SlSHN1* over-expression can improve tolerance of tomato plant to drought stress, the coding sequence (CDS) of *SlSHN1* was cloned in a binary plasmid under the control of the *CaMV 35S* constitutive promoter and transgenic tomato plants were generated. Transgenic tomato plants over-expressing the targeted gene were identified and analyzed for *SlSHN1* gene expression levels by using quantitative RT-PCR analysis (Figure S1). Two positive transgenic tomato lines with a single transgene event and expressing high levels of *SlSHN1* were selected for further analysis. The over-expression of *SlSHN1* in tomato resulted in growth retardation phenotypes where the transgenic plants were shorter than wild type and had a shiny appearance and dark green colored leaves (Figure 3a–c).

These observations are in general agreement with previous studies where the constitutive over-expression of the *SlSHN1* ortholog resulted in growth retardation in *Arabidopsis* plants [24,28,29]. However, the over-expression *SlSHN1* in tomato did not result in the formation of downward folded leaves as reported previously in *Arabidopsis* plant (Figure 3b,c). Furthermore, the severity of growth retardation phenotype in transgenic lines over-expressing *SlSHN1* was considerably milder when compared with tomato lines over-expressing the *SlSHN3* gene [32]. In general, the transgenic lines over-expressing *SlSHN1* behaved similarly to transgenic alfalfa plants over-expressing the *WXP1* gene, the *M. truncatula* ortholog closest to *WIN1/SHN1* [40].

To investigate the changes in leaf color in transgenic lines, chlorophyll content was determined and compared to wild type leaves. The over-expression of *SlSHN1* significantly increased the Chla, Chlb and total chlorophyll pigment concentrations in transgenic tomato plants when compared with the wild type plants (Figure 3d). These dark colored phenotypes might indicate lower endogenous GA levels in *SlSHN1* transgenic lines as observed previously in transgenic tomato lines over-expressing *SlDREB* (encoding AP2 domain transcription factor; [42]) and the *Arabidopsis Gibberellin methyl transferase 1* genes [43]. Furthermore, the over-expression of the *OsWR1* (*WIN1*/*SHN1* ortholog in rice) showed less chlorophyll leaching in drought stressed plants when compared with non-transgenic rice plants [36].

**Figure 4:**
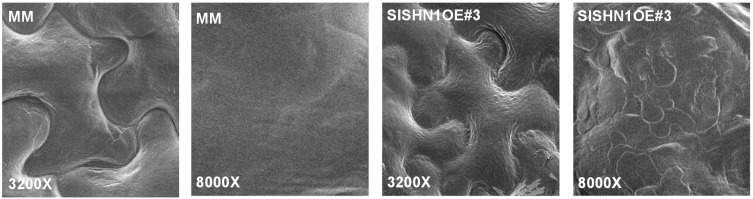
Scanning electron microscopy photos showing the leaf abaxial side in wild type (MM) and transgenic line over-expressing *SlSHN1* (SLSHN1OE#3) at two magnification powers (3200× and 8000×).

In addition, the glossy appearance of the leaves indicates that the over-expression of *SlSHN1* in tomato plant affected the leaf epidermis and its cuticular properties. For this purpose, the leaf abaxial side of transgenic lines over-expressing *SlSHN1* was observed by scanning electron microscopy and compared with wild type plants (Figure 4). In general the shape of the epidermal cells was similar between the wild type and transgenic lines over-expressing *SlSHN1* (Figure 4), however, the amount of deposited wax was higher in the transgenic lines when compared with the wild type plants (Figure 4). These higher amounts of wax deposition on leaf surface of *SlSHN1* transgenic lines were similar to the results obtained with transgenic *Arabidopsis* plants over-expressing *WIN1*/*SHN1* [24,25,29], transgenic alfalfa plants expressing the *M. truncatula WXP1* gene [40], transgenic rice plants over-expressing *OsWR1* [36] and transgenic *Arabidopsis* plants over-expressing *SlSHN3* [28].

**Figure 5:**
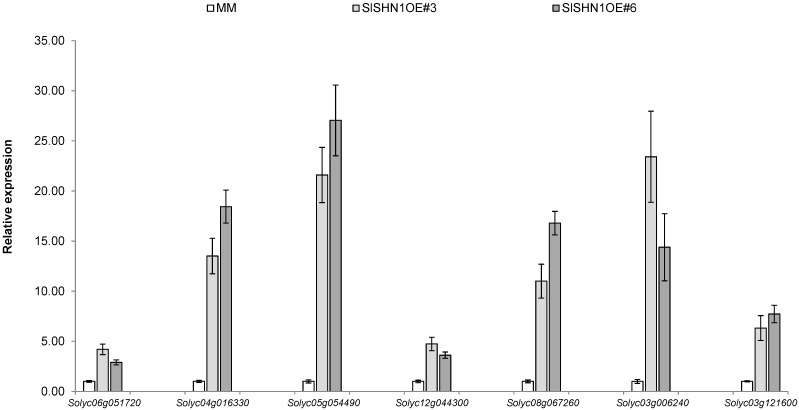
Gene expression analysis of seven cutin-related synthesis genes (*Solyc06g051720* (GDSL-motif lipase), *Solyc04g016330* (GDSL-motif lipase), *Solyc05g054490* (Enoyl-CoA reductase), *Solyc12g044300* (acyl-CoA synthase), *Solyc08g067260* (Fiddlehead), *Solyc03g006240* (HOTHEAD-like) and *Solyc03g121600* (HOTHEAD-like)) in leaves of transgenic line over-expressing *SlSHN1* (SlSHN1OE#3 and SlSHN1OE#6) and wild type plants (MM). The bars are standard deviations (SD) of three technical repeats.

To correlate the increase in wax deposition on the epidermis of transgenic lines over-expressing *SlSHN1* with the expression of tomato genes involved in the cutin pathway, 16 tomato cutin synthesis-related genes (Table S1) previously described to be up-regulated in tomato fruit epidermis tissue [44] were selected for gene expression analysis experiments. Gene expression analysis revealed that out of 16 targeted cutin synthesis-related genes, seven genes showed more than three-fold up-regulation in *SlSHN1* transgenic lines when compared with wild type plants (Figure 5). These genes include *Solyc06g051720* (encodes Gly–Asp–Ser–Leu (GDSL) motif lipase), *Solyc04g016330* (encodes Glycerol-3-P dehydrogenase), *Solyc05g054490* (Enoyl-CoA reductase), *Solyc12g044300* (encodes Acyl-CoA synthetase), *Solyc08g067260* (encodes putative Fiddlehead), *Solyc03g006240* (encodes putative HOTHEAD protein), and *Solyc03g121600* (encodes putative HOTHEAD protein). These results indicate that *SlSHN1* is involved, either directly or indirectly, in modulating the expression of cutin synthesis-related genes in tomato. Similarly, the over-expression of *WIN1*/*SHN1* in the *Arabidopsis* plant was found to regulate the expression of cutin synthesis-related genes such as the *GDSL-motif lipase* and *fatty acyl-CoA reductases* as possible downstream targets [27]. Furthermore, the silencing of the *SlSHN3* gene in tomato plants was associated with the down-regulation of genes encoding GSDL-motif lipases, putative acyltransferases and acyl-CoA synthetase, which overlaps with the *SlSHN1* targets in this study [28]. The over-expression of *OsWR1* in rice plants up-regulated the expression of several cutin-related synthesis genes including *OsHTH*-like, *OsGDSL* and *OsLACS2*-2 that showed similarity to up-regulated genes in this study.

**Figure 6:**
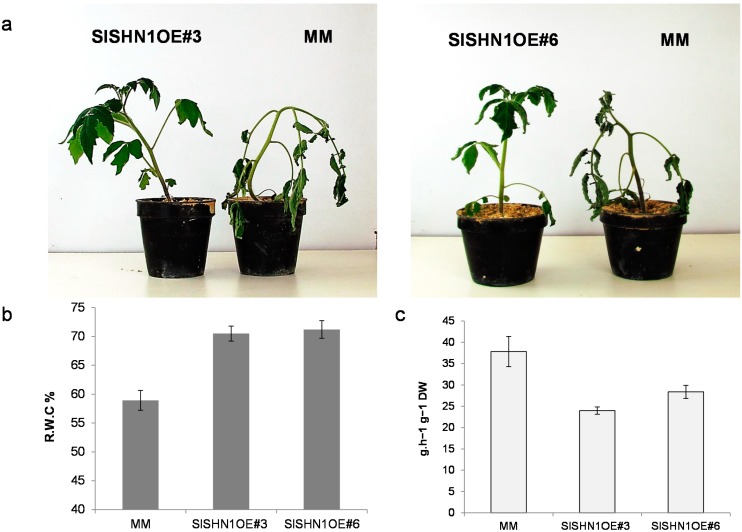
(**a**) Growth performance of tomato plants of two transgenic lines over-expressing *SlSHN1* (*SlSHN1OE#3* and *SlSHN1OE#6*) and wild type (MM) after 21 days of water withholding; (**b**) R.W.C. in leaves of two transgenic lines over-expressing *SlSHN1* (*SlSHN1OE#3* and *SlSHN1OE#6*) and wild type (MM) after 21 days of water withholding. Values are the means ± S.E. of 5 plants; (**c**) Water loss as measured by decrease in fresh weight after 2 h in detached leaves from two transgenic lines over-expressing *SlSHN1* (*SlSHN1OE#3* and *SlHN1OE#6*) and wild type (MM). Values are the means ± S.E. of 5 plants.

To analyze drought tolerance in the transgenic lines over-expressing *SlSHN1*, two-weeks old transgenic and wild type (included as control) seedlings growing in sand culture were subjected to water withholding condition for 21 days. The stress-subjected plants were monitored for growth and wilting behavior at the end of treatment. Under water deficit conditions, all wild type plants showed a wilted phenotype indicating that they suffered adversely from such conditions (Figure 6a). However, transgenic tomato plants over-expressing the *SlSHN1* gene showed enhanced tolerance to drought conditions and delayed wilting when compared with wild type plants (Figure 6a). Relative water content (R.W.C.) measurement of the stressed transgenic plants showed higher values when compared with wild type plants indicating a better water status in cells of transgenic lines (Figure 6b).

To test the effect of drought stress on water loss rate, fully expanded wild type and *SlSHN1* transgenic line leaves were detached from well-watered plants and subjected to dehydration for 2 h as described in the Experimental Section. After 2 h of dehydration, the detached leaves of the SlSHN1 transgenic lines lost significantly less water when compared with wild type leaves (Figure 6c). These results indicate that the *SlSHN1* transgenic lines showed improved tolerance to drought conditions when compared with wild-type plants. The drought tolerance behavior of the *SlSHN1* transgenic lines was similar to that of transgenic lines of its orthologous genes in *Arabidopsis* [24,25], rice [36] and alfalfa [40]. This drought tolerance behavior could be attributed to increased cuticular wax accumulation in *SlSHN1* transgenic lines and its possible effect on reducing water loss from epidermal tissue [28].

## 3. Experimental Section

### 3.1. Cloning of SlSHN1 in Tomato

Based on the amino acid sequence encoded in the previously described *WIN1/SHN1* gene in *Arabidopsis* (GenBank accession number: AAR20494.1), a TBLASTN search was performed against the annotated ITAG2.3 predicted tomato cDNA sequences database [33]. Using this approach, the most similar cDNA sequence to *WIN1/SHN1* gene was retrieved and was found to encode the tomato protein Solyc03g116610 described as ethylene-responsive transcription factor (GenBank accession number: XM_004235917). The identified gene was designated *SlSHN1* as described previously [28]. Using the cDNA sequence of *Solyc03g116610*, a gene-specific primer pair of 5'-ATGGTACAGGCAAAGAAGTTCAG-3' (*SlSHN1fwd*) and 5'-TAATTCCTGTTGAGGAGTTCCTC-3' (*SlSHN1rev*) was designed in order to isolate the full length CDS of *SlSHN1*.

To isolate the full length CDS of the *SlSHN1* gene, leaf tissue was harvested from two weeks old tomato cv. Money Maker seedlings and flash frozen in liquid nitrogen until total RNA extraction. Total RNA was isolated from leaf tissue using the SV Total RNA Isolation System Kit (Promega, Madison, WI, USA) following the manufacturer’s instructions. The isolated RNA was used to synthesize first strand cDNA library using the SuperScript^®^ First-Strand Synthesis System (Invitrogen, Carlsbad, CA, USA) and oligo T_(18)_ primer following the manufacturer’s instructions. The full-length CDS of tomato *SlSHN1* was amplified using PCR in a 25 μL reaction mixture containing 5 μL of cDNA as a template, 2.5 µL of dNTPs (100 µM), 5 μL of 5× PCR buffer, 0.5 µM of each primer and 0.25 µL of 5 U/µL GoTaq DNA polymerase (Promega, Madison, WI, USA). The PCR conditions were 94 °C for 5 min, followed by 40 cycles of 94 °C for 1 min, 55 °C for 1 min, and 72 °C for 1 min, and a final 10 min extension at 72 °C. The amplified PCR products were separated in 1% agarose gel stained with ethidium bromide. Positive PCR products were extracted from agarose gel using Wizard^®^ SV Gel and PCR Clean-Up System (Promega) and cloned into pGEM^®^-T Easy Vector System (Promega) following manufacturer’s instructions. Positive recombinant plasmids that contained the full-length *SlSHN1* cDNA were fully sequenced using the M13 reverse and forward sequencing primers by ABI 3730XL machine by Macrogen (Seoul, Korea).

### 3.2. Bioinformatics Analysis

Sequence analysis, chromosomal location and annotation predication was performed using Sol Genomic Network [34,45] and MIPS tomato genome database [35]. For multiple sequence alignments analysis, the Clustal_X program (version 2.0) [46] was used. A phylogenetic analysis was carried out using MEGA6 [46]. The protein sequence of SlSHN1, together with ERF paralogues from tomato and *Arabidopsis* belonging to group V [38] were retrieved from phytozome databases [47] and aligned using the ClustalW algorithm in the MEGA6 [48]. The alignment was used to calculate distance matrices for neighbor-joining analyses with the Kimura two-parameter model. Bootstrap analysis with 10,000 replicates was performed to test the robustness of the internal branches. For promoter analysis, PlantCARE [41] database was used to identify stress-related *cis*-regulatory elements.

### 3.3. Plant Material, Growth Conditions and Stress Treatments

Tomato cultivar “Moneymaker” was used for the gene expression analysis experiments in response to drought treatment. Transgenic tomato lines over-expressing *SlSHN1* gene were used for performance analysis under drought conditions in comparison with Moneymaker plants. For transgenic plants generation, the full length CDS of *SlSHN1* was introduced into the binary plasmid pCABIMA1302 by replacing the *GFP* gene at the *Nco*I and *Bst*EII sites. The introduced *SlSHN1* CDS was under the control of *CaMV 35S* promoter. Positive binary plasmids containing *SlSHN1* CDS were used for *Agrobacterium* tumefaciens-mediated transformation of the Moneymaker cultivar at the Ralph M. Parsons Foundation Plant Transformation Facility at University of Califorlia Davis. Transgenic seeds from T1 plants were selected on MS medium containing 50 mg/L kanamycin and lines showing 3:1 segregation for the antibiotic resistance were selected to get the T2 progeny plants. T2 plants seeds were further analyzed for transgene existence using PCR, segregation for antibiotic resistance plants and gene expression levels using RT-qPCR analysis using first strand cDNA library prepared from total RNA isolated transgenic plants as described below. In addition, transgene copy number was determined using RT-qPCR using *neomycin phosphoryl-transferase II* (*nptII*) gene specific primers and the *SlActin* (*Solyc03g078400*) gene (Table S1) as internal control as described previously [49]. Two T1 plants having single copy of the transgene and showing high level of *SlSHN1* expression were selected to obtain T2 seeds by screening for antibiotic resistance plants as described in Wang and Waterhouse [50]. T2 homozygous plants from both lines were further selected and then used for the stress experiments.

For gene expression experiments, tomato cv. Moneymaker seeds were soaked in water for 2 days at 25 °C and then washed with sterilized water before sowing into small pots (10 cm diameter × 10 cm depth) filled with acid-washed sand. After germination, tomato seedlings were placed under controlled conditions (continuous 25 °C temperature, photoperiod of 16 h light/8 h dark with 80 µmol·m^−2^·s^−1^ photon flux density) and irrigated daily with fixed volume of Hoagland solution. For drought treatment, two weeks old tomato seedlings were subjected to water withholding for 3, 5 and 7 days. For each treatment, three replicates were used and for each replicate young leaves were harvested from three plants.

For transgenic lines performance under stress conditions, two weeks old seedlings of wild type and transgenic lines grown under controlled conditions were subjected to drought conditions by water withholding for 21 days and the wilting behavior of the treated plants was monitored.

### 3.4. Physiological Measurements

For physiological measurements, leaf relative water content was measured under control (well-watered plants) and drought stress conditions (after 21 days of water withholding) for transgenic and non-transformed Moneymaker plants. For this purpose, fully expanded leaves were taken from transgenic and wild-type plants and their fresh weights were immediately recorded after leaf excision. The excised leaves were soaked in distilled water for 6 h at room temperature in darkness and the turgid weight (TW) was recorded. Total dry weight (DW) was then recorded after drying for 24 h at 70 °C. R.W.C. was calculated according to Barrs and Weatherley [51]: R.W.C. (%) = [(FW − DW)/(TW − DW)] × 100%. For water loss rate (WLR) experiments, fully expanded leaf was excised from the four weeks old well-watered transgenic lines and non-transformed Moneymaker plant and immediately weighed (T0). The excised leaves were then left on filter paper for 2 h and weighed (T2). Total dry weight (DW) was then recorded after drying for 24 h at 70 °C and the WLR was measured according to Ristic and Jenks [52]: WLR (g·h^−1^·g^−1^·DW) = [(*F*_T0_ − *F*_T2_) × 60]/[DW × (T2 − T0)]. To measure the chlorophyll content (Chla, Chlb and total Chl), excised leaves from well-watered transgenic lines and non-transformed Moneymaker plants were used following a modified protocol as described previously [53].

### 3.5. Scanning Electron Microscopy (SEM)

Leaf fragments from transgenic tomato and wild type were plants first fixed in 5% glutaraldehyde and mounted on stubs for coating. Thereafter, the leaf samples were coated with platinum using a sputter coater (Emitech K550X, Quorum Technology Ltd., Laughton, UK). The coated samples were then transferred to a scanning electron microscope (FEI inspect F50, FEI, Tokyo, Japan) for examination.

### 3.6. Quantitative Real-Time RT-PCR (qRT-PCR) Analysis

For quantitative real-time PCR (qRT-PCR) analysis of stress-treated Moneymaker plants, total RNA was isolated from leaf samples taken from treated plants as described above. Gene-specific primers pairs for the *SlSHN1* gene, the stress-inducible *Le16* gene [39] and *Solyc03g078400* (encoding actin, a house-keeping gene used as an internal reference control for relative gene expression analysis) were designed (Table S1). The amplification of the targeted genes were carried out using the GoTaq^®^ qPCR Master Mix Kit (Promega), and the real-time detection of products was performed in a Mini-Opticon Real Time PCR System (BioRad, Hercules, CA, USA). All cDNA samples were analyzed in triplicate, and the cDNA was derived from at least two biological replicates. Thermal cycling conditions consisted of 40 cycles of 94 °C for 30 s, 58 °C for 30 s and 72 °C for 45 s, plus a final extension at 72 °C for 5 min. The relative changes in gene expression were quantified as described in Vandesompele *et al.* [54].

For gene expression analysis of putative wax biosynthesis genes in *SlSHN1* over-expressing transgenic and Moneymaker plants, total RNA was extracted from leaf tissue from two weeks old sand-grown plants using a SV Total RNA Isolation System Kit (Promega) following the manufacturer’s instructions. Gene-specific primers pairs for 16 selected putative wax biosynthesis genes (described in [44]) were designed using Primer 3 Software ([55]; Table S1). The qRT-PCR analysis was performed as described above.

## 4. Conclusions

In summary, *SlSHN1*, the closest ortholog to WIN1/SHN1, is a transcriptional activator of wax/cutin synthesis and its over-expression in tomato plant resulted in improving drought tolerance.

## Supplementary Materials

Supplementary materials can be found at: http://www.mdpi.com/1422-0067/15/11/19499/s1.
